# Comparative transcriptome analysis reveals key genes associated with pigmentation in radish (*Raphanus sativus* L.) skin and flesh

**DOI:** 10.1038/s41598-021-90633-5

**Published:** 2021-06-01

**Authors:** Jifang Zhang, Jian Zhao, Qunyun Tan, Xiaojun Qiu, Shiyong Mei

**Affiliations:** 1 Institute of Bast Fiber Crops, Chinese Academy of Agricultural Science, Changsha, China; 2 Center for Southern Economic Crops, Chinese Academy of Agricultural Science, Changsha, China; 3 Novogene Bioinformatics Institute, Beijing, China

**Keywords:** Molecular biology, Plant sciences

## Abstract

Radish (*Raphanus sativus*) is an important vegetable worldwide that exhibits different flesh and skin colors. The anthocyanins responsible for the red and purple coloring in radishes possess nutritional value and pharmaceutical potential. To explore the structural and regulatory networks related to anthocyanin biosynthesis and identify key genes, we performed comparative transcriptome analyses of the skin and flesh of six colored radish accessions. The transcript profiles showed that each accession had a species-specific transcript profile. For radish pigmentation accumulation, the expression levels of anthocyanin biosynthetic genes (*RsTT4*, *RsC4H*, *RsTT7*, *RsCCOAMT*, *RsDFR*, and *RsLDOX*) were significantly upregulated in the red- and purple-colored accessions, but were downregulated or absent in the white and black accessions. The correlation test, combined with metabolome (PCC > 0.95), revealed five structural genes (*RsTT4*, *RsDFR*, *RsCCOAMT*, *RsF3H*, and *RsBG8L*) and three transcription factors (*RsTT8-1*, *RsTT8-2*, and *RsPAR1*) to be significantly correlated with flavonoids in the skin of the taproot. Four structural genes (*RsBG8L*, *RsDFR*, *RsCCOAMT*, and *RsLDOX*) and nine transcription factors (*RsTT8-1*, *RsTT8-2*, *RsMYB24L*, *RsbHLH57*, *RsPAR2L*, *RsbHLH113L*, *RsOGR3L*, *RsMYB24,* and *RsMYB34L*) were found to be significantly correlated with metabolites in the flesh of the taproot. This study provides a foundation for future studies on the gene functions and genetic diversity of radish pigmentation and should aid in the cultivation of new valuable radish varieties.

## Introduction

Flavonoids are plant polyphenolic secondary metabolites that are widespread in the plant kingdom. Flavonoids can be classified into many subgroups based on their chemical structures and modifications, including flavanones, flavones, isoflavonoids, flavanols, anthocyanins, and flavonols^1^. Anthocyanins are the major pigment metabolites of flavonoid compounds. They are abundant in the flowers, fruits, seeds, and leaves of many plant species^2^ and play important roles in plant protection and reproduction^3,4^. They can help plants attract pollinators and seed dispersers by stimulating red, purple, and blue pigments in plant tissues^3^. In recent years, there has been increased interest in anthocyanins because of their wide use as natural commercial food pigments and their potential health benefits^5–7^.

Flavonoid biosynthesis and regulatory pathways have been extensively described. Enzymes, such as CHS, F3H, F3′H, F3′5′H, and LDOX/ANS, are involved in flavonoid biosynthesis and may act as metabolic hubs that influence the overall efficiency, specificity, and regulation of these pathways^8–10^. DNA-binding R2R3 MYB transcription factors (TFs), MYC-like basic helix-loop-helix (bHLH), and WD40-repeat proteins interact in the regulation of flavonoid biosynthesis^11–19^. Plant hormones^20–22^ and environmental factors^23–26^ also play important roles in the regulation of anthocyanin biosynthesis. Nevertheless, the mechanisms that regulate anthocyanin metabolism in different plant species and tissues require further investigation^27^.

Radishes (*Raphanus sativus*) are common vegetables worldwide that possess a wide variety of colors, shapes, and sizes. The pigments in radish taproots have health-promoting properties. Based on anthocyanin production, radishes can be categorized into those that have anthocyanins in their skin and flesh, and those that have anthocyanins only in their skin. Several varieties with different skin and/or flesh taproot colors (e.g., white, green, red, purple, and black) are widely cultivated and consumed. However, the mechanisms that regulate anthocyanin catabolism in radish have not received much research attention.

Fortunately, correlation and clustering analyses based on transcript and metabolite information have been used to further build networks between genes and metabolites in many plants, including *Arabidopsis*^28,29^, tomato (*Solanum lycopersicum*)^30–32^, potato (*Solanum tuberosum*)^33–34^, grape (*Vitis vinifera*)^27^, and *Actinidia arguta*^35^. The integration of “omics” datasets (i.e., transcriptomes and metabolomes) is considered a useful approach for discovering and identifying potential genes that regulate the determination of radish pigmentation.

In this study, RNA sequencing (RNA-seq) analysis using the Illumina platform was used to study the genetic control of the differential accumulation of anthocyanins. By combining our previous metabolite datasets^36^ in a bioinformatics analysis, the major metabolic pathways related to radish taproot pigmentation were deduced, and candidate genes targeting the regulation of radish pigmentation were examined.

## Results

### Anthocyanin contents of radish taproot skin and flesh

**Figure 1:**
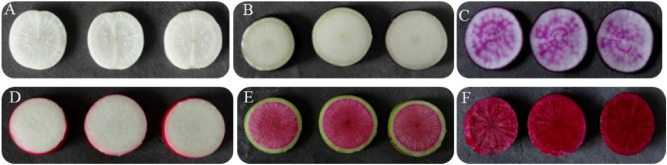
Taproot colors of the six radish accessions: (**A**) Shizhuangbai (SZB) has white taproot skin (WS) and white flesh (WF); (**B**) black radish has black taproot skin (BS) and white flesh (WF); (**C**) Zijinling (ZJL) has purple taproot skin (ZJLS) and purple flesh (ZJLF); (**D**) Manshenhong (MSH) has light red taproot skin (PS) and white flesh (WF); (**E**) Xinlimei has green taproot skin (XLMS) and red flesh (XLMF); (**F**) Touxinhong (TXH) has dark red taproot skin (RS) and dark red flesh (RF).

**Figure 2:**
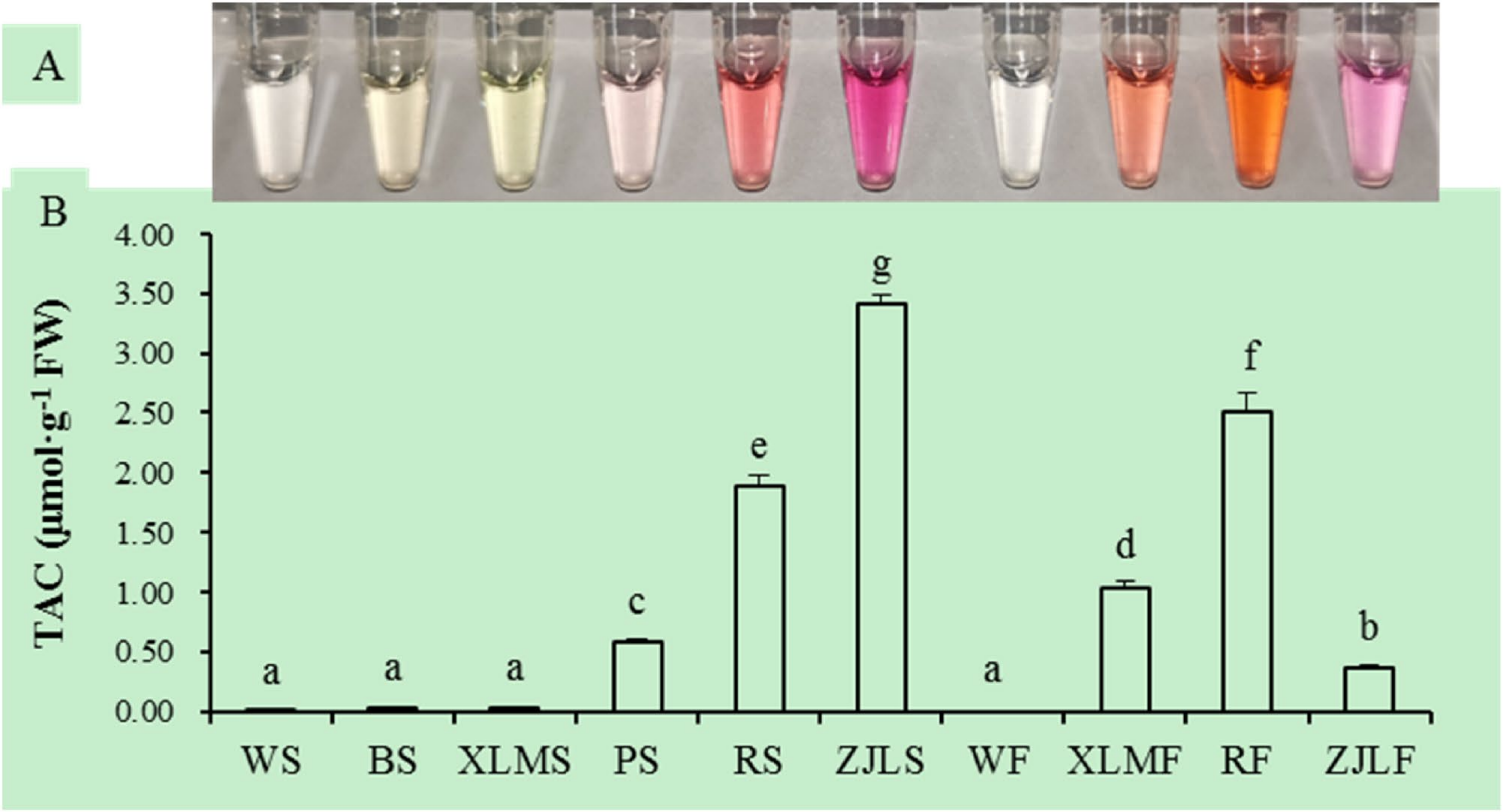
Pigment contents in the skin and flesh of radish taproots. (**A**) The color of the supernatant of the sample extracts. (**B**) Total anthocyanin content of the skin and flesh of radish taproots.

Six radish accessions (Fig. 1) with various skin and flesh colors were used for the anthocyanin content assay. The skin of the radish taproot (Fig. 2), ZJL, which is purple in color, exhibited the highest anthocyanin content, followed by the dark red TXH and the light red MSH. The skin of SZB, black radish, and XLM exhibited trace or undetectable contents. In terms of flesh color (Fig. 2), TXH, which has dark red flesh, showed the highest anthocyanin content, followed by the red-fleshed XLM and the purple-fleshed ZJL. The white flesh of SZB had no detectable anthocyanin content. The purple skin of ZJL had a higher anthocyanin content than the dark red flesh of TXH.

### RNA sequencing and assembly

The six radish accessions (Fig. 1) with various skin and flesh colors were passed through RNA-seq and analysis, generating a total of 1,695,680,738 raw reads (Table 1). After adaptor sequence trimming and filtering out of low-quality reads, 1671,174,278 (98.55%) clean reads were obtained with an average GC content of 46.74% and a Q20 average base quality score of 97.79%. A total of 250.71 Gb of high-quality reads were obtained for the radish samples. Of these clean reads, 68.62–87.7% were mapped to the reference genome WK10039, and 62.3–80.24% were uniquely mapped to WK10039. Furthermore, 31.15–40.09% of the clean reads were mapped to the positive strands, and 31.14–40.15% were mapped to the negative strands (Table S2).

**Table 1:** Details of the raw data and clean data of all radish samples of various colors.

Sample	Raw reads	Clean reads	Clean bases (G)	Q20avg (%)	GC_pct (%)
WST	176,281,664	173,933,900	26.1	97.79	46.74
WFT	177,066,062	174,510,406	26.18		
RST	168,928,392	166,285,974	24.95		
RFT	168,355,314	165,648,598	24.85		
BST	170,937,186	168,309,646	25.25		
XLMST	163,260,394	160,683,094	24.1		
XLMFT	163,327,024	160,571,396	24.09		
PST	154,019,884	151,985,018	22.8		
ZJLST	167,115,082	165,168,530	24.77		
ZJLFT	186,389,736	184,077,716	27.62		
Total	1,695,680,738	1,671,174,278	250.71	_	_

*WST* transcript data of SZB with white taproot skin, *WST* transcript data of SZB with white flesh, *RST* transcript data of TXH with dark red taproot skin, *RFT* transcript data of TXH with dark red flesh, *BST* transcript data of black radish, *XLMST* transcript data of Xinlimei with green taproot skin, *XLMFT* transcript data of Xinlimei with red taproot flesh, *PST* transcript data of MSH with light-red taproot skin, *ZJLST* transcript data of ZJL taproot skin, *ZJLFT* transcript data of ZJL taproot flesh.

### Differential gene expression levels

**Figure 3:**
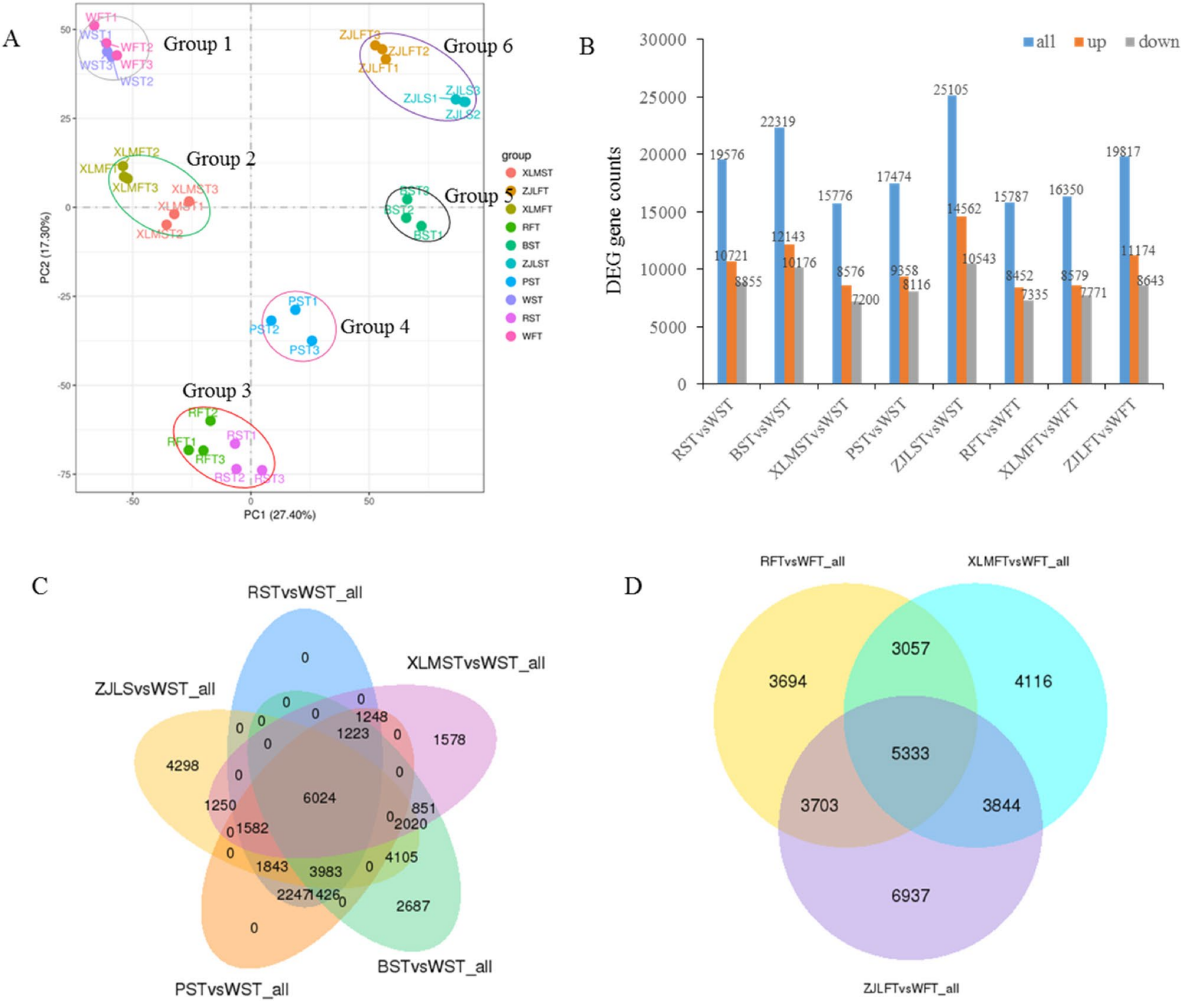
PCA and distribution of DEGs based on changes in the color of radish taproot skin and flesh. (**A**) PCA of the relative differences in the RNA-seq profiles among the different radish accessions. The sampling groups are color-coded as follows: pink-purple, SZB (WST and WFT); green–red XLM (XLMST and XLMFT); green-pink, TXH (RST and RFT); green, black radish (BST); blue-brown, ZJL (ZJLST and ZJLFT); blue, PST. (**B**) The numbers of up- and downregulated DEGs. (**C**) Venn diagram showing the number of DEGs revealed by pairwise comparisons in skin color. (**D**) Venn diagram showing the number of DEGs revealed by pairwise comparisons in flesh color. (**A**,**C**,**D**) were produced in R3.0.3 (https://www.r-project.org/).

Principal component analysis (PCA) was used to assess inter-group differences and sample duplication within groups. In the PCA plot, 10 samples from the six accessions clustered into six independent groups, suggesting that each group had a relatively distinct transcript profile (Fig. 3A). Group 1 included white-skinned and white-fleshed taproots of the SZB accession (WST and WFT), and group 2 included green-skinned and red-fleshed taproots of the XLM accession (XLMST and XLMFT). The dark red-skinned and dark-red-fleshed taproots of the TXH accessions (RST and RFT) clustered in group 3, and the purple-skinned and purple-fleshed taproots of the ZJL accessions (ZJLST and ZJLFT) clustered in group 6. Accessions with the same colored skin and flesh clustered together.

To understand the molecular basis and predict the candidate genes responsible for color polymorphisms in radish, differentially expressed genes (DEGs) were identified by pairwise comparisons of the expression levels between colored and white samples. The transcriptome analysis revealed that 19,576 (10,721 upregulated and 8855 downregulated), 22,319 (12,143 upregulated and 10,176 downregulated), 15,776 (8576 upregulated and 7200 downregulated), 17,474 (9358 upregulated and 8116 downregulated), and 25,105 (14,562 upregulated and 10,543 downregulated) DEGs had at least a two-fold change in the skin of TXH, black radish, XLM, MSH, and ZJL, respectively, compared to SZB (Fig. 3B). There were 15,787 (8452 upregulated and 7335 downregulated), 16,350 (8579 upregulated and 7771 downregulated), and 19,817 (11,174 upregulated and 8643 downregulated) transcripts with at least a two-fold change in the flesh of TXH, XLM, and ZJL, respectively, compared to SZB (Fig. 3B). Additionally, the Venn diagram analysis showed that 6024 and 5333 DEGs were considerably differentially expressed in the skin and flesh in the pairwise comparisons, respectively (Fig. 3C,D).

A Kyoto Encyclopedia of Genes and Genomes (KEGG)^37^ enrichment analysis was conducted to identify pathway-related DEGs in the colored skin and flesh of radish. A total of 111 pathways were enriched (Supplementary Table S3). The “biosynthesis of amino acids”, “glyoxylate and dicarboxylate metabolism”, “flavonoid biosynthesis”, “photosynthesis-antenna proteins”, “glycine, serine, and threonine metabolism”, and “carbon metabolism” pathways were significantly enriched in RS vs. WS (Fig. S1A). The “plant-pathogen interaction” pathway was significantly enriched in the skin of MSH (Fig. S1B). The “cyanoamino acid metabolism”, “glyoxylate and dicarboxylate metabolism”, “carbon fixation in photosynthetic organisms”, and “carbon metabolism” pathways were significantly enriched in the skin of XLM (Fig. S1D). DEGs related to the “ribosome” and “ribosome biogenesis in eukaryotes” pathways were significantly enriched in the flesh of XLM (Fig. S1G).

### Correlation analysis between transcripts and flavonoid derivatives revealed a differential network of flavonoid biosynthesis

**Figure 4:**
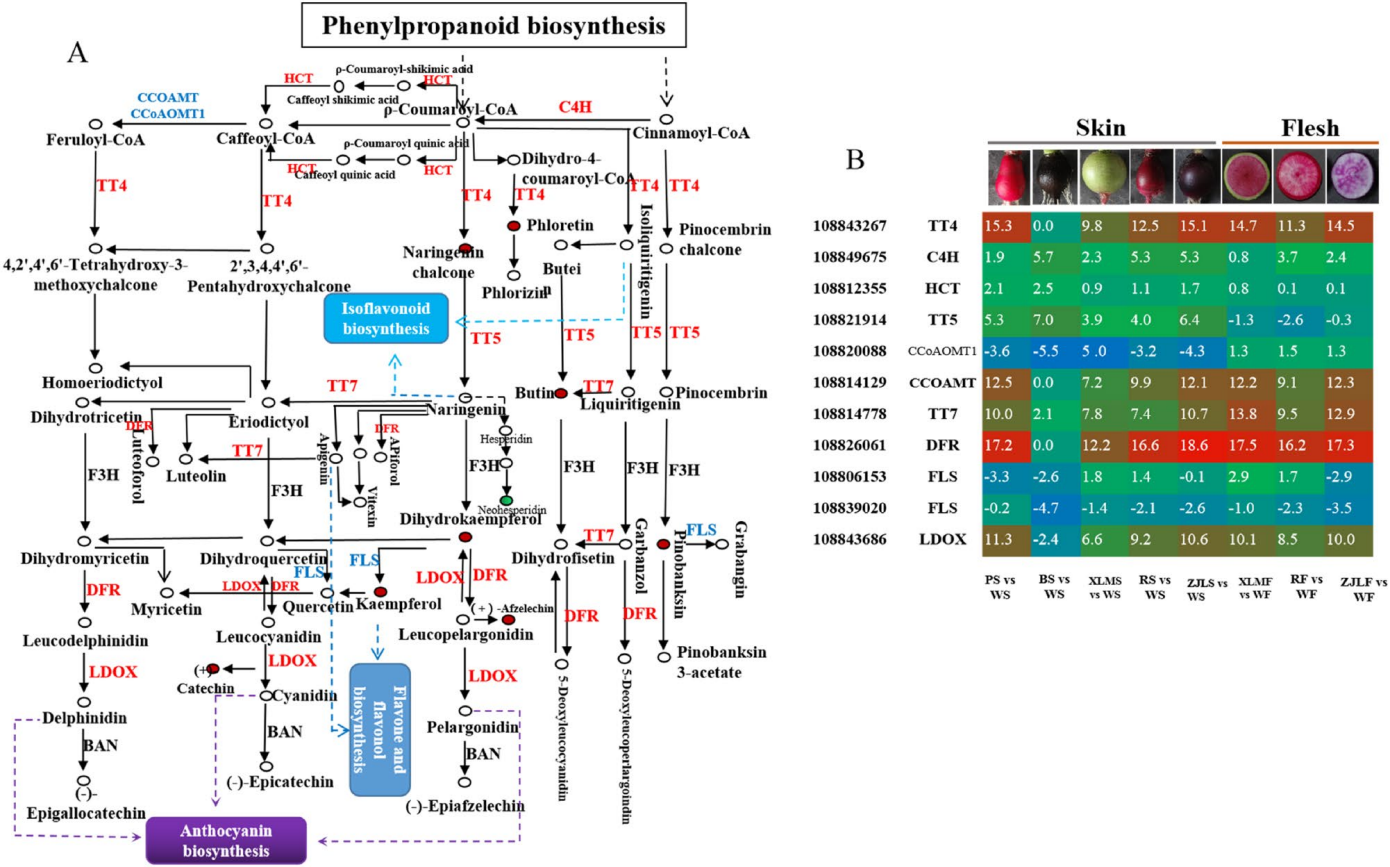
Biosynthetic pathway of flavonoids and the enriched or significantly expressed genes involved in the pathway. (**A**) The flavonoid biosynthesis pathway was constructed based on the KEGG pathway (ko00941)^37^ and previous works. The genes shown in red were upregulated in samples, while genes shown in blue color were downregulated. Red circles indicate upregulated metabolites, and green circles indicate downregulated metabolites. (**B**) Expression patterns of the genes involved in flavonoid biosynthesis compared to WS. *TT4* chalcone synthase, *C4H* cinnamate-4-hydroxylase, *HCT* shikimate O-hydroxycinnamoyltransferase, *TT5* chalcone isomerase, *TT7* flavonoid 3'-monooxygenase, *CCoAOMT1* caffeoyl coenzyme A methyltransferase, *CCOAMT* caffeoyl-CoA O-methyltransferase, *DFR* dihydroflavonol-4-reductase, *FLS* flavonol synthase, *LDOX* anthocyanidin synthase, *F3H* 108852668, flavanone 3-hydroxylase, *BAN* anthocyanidin reductase.

To understand the network of flavonoids uncovered by the differential distribution of flavonoid derivatives between colored and white radishes, we conducted correlation tests between the quantitative changes in the metabolites and transcripts. A total of 11 structural genes were enriched in the red and purple radishes compared to the white and black radishes (Fig. 4A,B). Seven genes (*RsTT4*,108843267; *RsC4H*,108849675; *RsHCT*,108812355; *RsTT7*,108814778; *RsCCOAMT*,108820088; *RsDFR*,108826061; and *RsLDOX*,108843686) were upregulated in the red and purple radishes, while two genes (*RsF3H* and *RsBAN*) did not change significantly. *RsFLS* (108839020) was downregulated in all of the samples. *RsTT5* (108821914) was upregulated in the skin of the five colored radishes, but was downregulated in the flesh of XLM, TXH, and ZJL.

**Figure 5:**
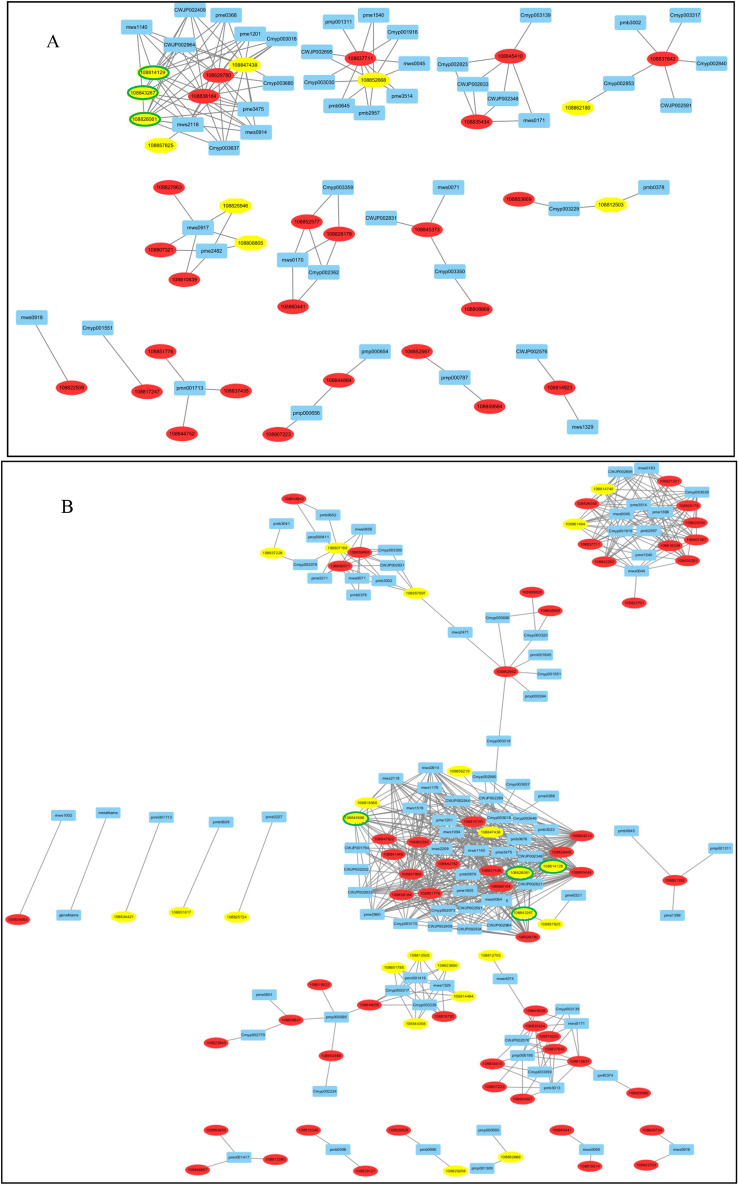
Connection network between the structural genes, TFs, and flavonoid-related metabolites. (**A**) Network between the genes and flavonoid metabolites in the taproot skin. (**B**) Network between the genes and flavonoid metabolites in the taproot flesh. Only genes with correlation coefficients > 0.95 are shown. Yellow circles with green frames represent the ID of structural genes involved in flavonoid biosynthesis; the other yellow circles represent the ID of structural genes involved in phenylpropanoid biosynthesis; the red circles indicate the TFs, and the blue boxes represent flavonoid-related metabolites. A and B were visualized using Cytoscape v2.8.2 (https://cytoscape.org/).

The correlation analysis between transcripts and flavonoid derivatives showed that different interaction modes were responsible for the color diversity of the taproot skin and flesh. For the taproot skin, three genes involved in flavonoid biosynthesis, seven genes involved in phenylpropanoid biosynthesis, and 35 TFs were found to be strongly correlated (Pearson’s correlation coefficient (PCC) > 0.95) with 48 metabolites (Fig. 5A, Table S4). A total of 11 flavonoids were found to be significantly correlated with *RsBG8L* (108847438, encodes beta-glucosidase 8-like), 10 flavonoids were found to be significantly correlated with *RsDFR* (108826061, encodes dihydroflavonol-4-reductase), 10 metabolites were found to be significantly correlated with *RsCCOAMT* (108814129, encodes caffeoyl-CoA O-methyltransferase), six metabolites were found to be significantly correlated with *RsTT4* (108843267, encodes chalcone synthase), and nine flavonoids were found to be significantly correlated with *RsF3H* (108852668, encodes flavanone 3-hydroxylase). Eleven flavonoids were found to be significantly correlated with *RsTT8-1* (108829780) and *RsTT8-2* (108838184) and nine metabolites were found to be significantly correlated with *RsPAR1*(108837711), indicating that these five structural genes and three TFs play critical roles in flavonoid biosynthesis in radish skins.

For taproot flesh, four structural genes were found to be involved in flavonoid biosynthesis, 20 genes were found to be involved in phenylpropanoid biosynthesis, and 57 TFs were strongly correlated (PCC > 0.95) with 93 metabolites (Fig. 5B, Table S5). A total of 29 flavonoids were found to be significantly closely correlated with RsBG8L, 25 flavonoids were found to be significantly closely correlated with *RsDFR*, 20 metabolites were found to be significantly correlated with *RsLDOX* (108843686, encodes anthocyanidin synthase), 16 metabolites were found to be significantly correlated with *RsCCOAMT*. 29 metabolites were found to be significantly correlated with *RsbHLH57* (10882212), 28 metabolites were found to be significantly correlated with *RsOGR3L* (1086444), and 26 metabolites were found to be significantly correlated with *RsbHLH113L* (108810191), *RsMYB24* (108828405), *RsMYB34L* (108852392), and *RsPAR2L* (108860104). Twenty-four flavonoids were found to be significantly correlated with *RsTT8-2*, and 23 flavonoids were found to be significantly correlated with *RsTT8-1* and *RsbHLH19* (108851776), suggesting that these four structural genes and nine TFs play important roles in flavonoid biosynthesis in radish flesh.

It has been reported that the overexpression of MdMYB24L results in higher anthocyanin content in transgenic apples than in the wild-type controls^38^. bHLH57 interacts with rdo5 to control ABA biosynthesis and seed dormancy in *Arabidopsis*^39^*,* and *AtbHLH19* plays roles in salt tolerance in *Arabidopsis*^40^. *TT8* has been well characterized in *Arabidopsis*^41,42^*, Brassica juncea*^43^, and radishes^44^. AtTT8, a positive regulator, controls its own expression in a feedback regulation involving TTG1 and homologous MYB and bHLH factors, allowing for the strong and cell-specific accumulation of flavonoids in *A. thaliana*^41,42^. Natural mutations in two homoeologous *TT8* genes control the yellow seed coat trait in *B. juncea*^43^. RsTT8 interacts with MYB1 to activate the promoter of *RsCHS* and *RsDFR* and so controls pigmentation accumulation in radishes^44^.

### Confirmation of the transcriptome data using real-time quantitative PCR (RT-qPCR)

**Figure 6:**
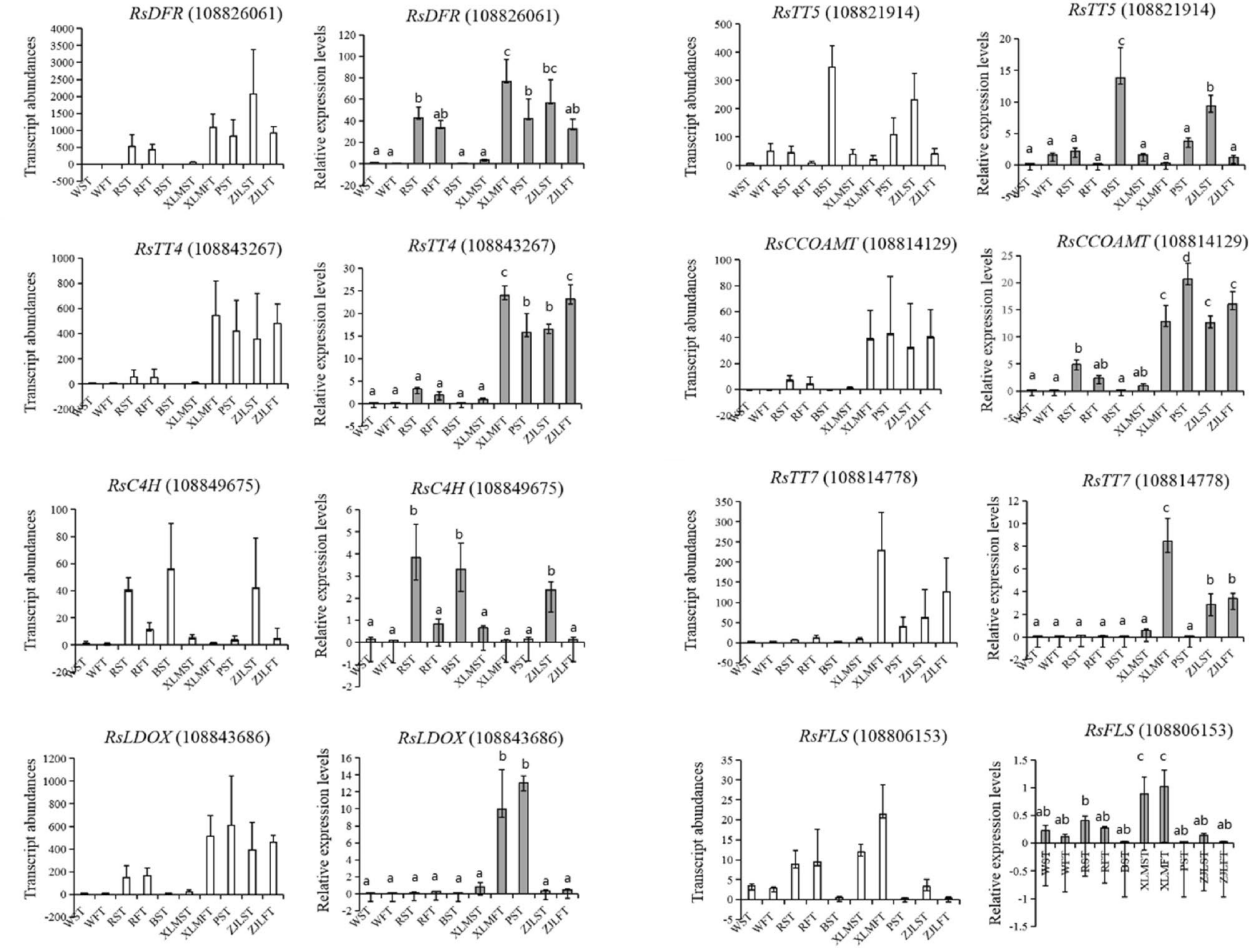
Validation of the transcriptomic data using RT-qPCR. Transcript abundances detected by transcriptome sequencing and expressed in FPKM are shown on the left. Relative expression levels detected by RT-qPCR and expressed in 2^−ΔΔCT^ are shown on the right. Data are presented as the mean of three replicates with three biological repeats. The significant differences among samples were evaluated by Dunnett’s test at *P* < 0.05.

Eight genes involved in flavonoid metabolism were selected for RT-qPCR assays to validate the transcriptome datasets obtained from RNA-seq. The results of these assays were consistent with the transcriptome analysis (Fig. 6).

## Discussion

Taproot skin and flesh color determine the appearance, quality, and nutritional value of radishes. To understand the molecular basis of the differential distribution of flavonoid derivatives between colored and white radishes, core genes in the flavonoid pathway were studied in detail. The results showed that most of the UniGenes demonstrated significant changes in expression level, regardless of whether they were in the early stage (RsTT4, chalcone synthase; RsC4H, cinnamate-4-hydroxylase; RsTT7, flavonoid 3'-monooxygenase; RsCCOAMT, caffeoyl-CoA O-methyltransferase) or late stage (RsDFR, dihydroflavonol-4-reductase; RsLDOX, anthocyanidin synthase), and showed higher transcript abundances in red and purple radishes than in the white radishes (Figs. 4, 5). Additionally, core genes involved in upstream phenylpropanoid biosynthesis also affect the productivity of the following flavonoid pathway. These genes, absent or undetectable, are thought to be flux-limiting genes that lead to colorant elimination in white radishes.

It is already known that TT4 catalyzes the first reaction in anthocyanin biosynthesis and helps form the intermediate chalcone, which is the primary precursor of all flavonoid classes^45^. Thus, if TT4 reactions are strongly constrained, then anthocyanin production and that of nearly all other flavonoids could be effectively eliminated^46^. DFR reduces dihydroflavonols to colorless leucoanthocyanidins (apigenin-related glycosides, leucocyanidin, and leucopelargonidin) that are subsequently catalyzed by LDOX to form cyanidin- and pelargonidin-related glycosides, which are then catalyzed by ANS into colored anthocyanidins^47^. Kaempferol-related glycosides are downstream of dihydrokaempferol, catalyzed by FLS, and fluxed into flavone and flavonol biosynthesis. *DFR* genes have been studied thoroughly in many plant species, including *Medicago truncatula*^48^, *Camellia sinensis* ^49^, *Ginkgo biloba* ^50^, *Populus trichocarpa* ^51^, and *Malus* crabapples^52,53^. Overexpression of *DFR* genes from *Rosa rugosa* and *Petunia hybrida* in white tobacco flowers promotes anthocyanin biosynthesis, corresponding to increased red pigmentation accumulation^54^. The altered expression of *DFR* can affect the expression of other anthocyanin biosynthesis genes^54^. The expression of *DFR* can also be positively and negatively affected by MYB TFs. For example, the apple TF MdMYB10 has been shown to regulate the red pigmentation in the fruit flesh, and MdMYB1 controls the color of fruit skin. They interact with bHLH genes to trans-activate the DFR promoter and increase red pigmentation^53^. TT8 acts a positive regulator of anthocyanin biosynthesis in *Arabidopsis* siliques and *Raphanus sativus*^41,42,44^. The expression of the AtMYB7 gene can repress the expression of DFR and UGT^55^.

In our study, the expression levels of *RsDFR*, *RsTT4*, *RsCCOAMT*, and *RsLODX* were undetectable in white radishes, but were abundant in the red-color radishes (Fig. 6). The correlation analysis verified that most flavonoids were significantly correlated with the transcript expression of *RsBG8L, RsCCOAMT*, and *RsDFR* both in the skin and in the flesh of the radish taproot. These three genes may lose their biocatalyst function and become pseudogenes due to variations in gene structure or regulation by correlated TFs, which were significantly associated with kaempferol-related glycosides. The radish basic helix-loop-helix TFs, *RsTT8-1* and *RsTT8-2,* were both significantly associated with flavonoids in the skin and flesh of taproot, indicating that these two TFs play crucial roles in the accumulation of pigments in the taproots. It has been reported that *RsTT8* interacts with *RsMYB1* to activate the promoters of *RsCHS* and *RsDFR*^44^, which confirmed the results of our correlation analysis. We here propose that the limitations of flux in upstream reactions and the multishunt process in downstream reactions could lead to the elimination of colored pigmentation in white radishes.

## Materials and methods

### Plant materials

Six advanced radish inbred lines were used in this study: Touxinhong (TXH), with dark red taproot skin and flesh; Xinlimei, with red taproot flesh and green skin; Shizhuangbai (SZB), with white taproot skin and flesh; black radish with black taproot skin and white flesh; Zijinling (ZJL), with purple taproot skin and flesh; and Manshenhong (MSH), with light red taproot skin and white flesh. All plants were grown in pots at the Changsha experimental station of the Institute of Best Fiber Crops, Chinese Academy of Agricultural Sciences, in April 2019. Our research complied with local guidelines and legislation, and the required permissions and licenses for the study were obtained. Developing taproots were harvested at 50 days after sowing. The skin and flesh of the taproots were separated using a blade on a clean table, sampled, and stored at − 80 °C for further analysis. Each sample included three biological repeats and each repeat included five individual plants.

### Total anthocyanin content measurement

Total anthocyanins were extracted using a plant anthocyanin content assay kit (Solarbio corporation, Beijing, China) with modifications. The samples of radish taproot skin and flesh were powdered, and 0.1 g of powder was placed into 1 mL of extraction buffer and incubated for 30 min at 60 °C. This was then centrifuged for 10 min at 12,000 rpm. The absorbance values (A530 and A700) of the supernatant reaction solution were measured using a microplate reader. All samples were evaluated in three independent biological replicates.

### RNA isolation and transcriptome sequencing

Total RNA of each sample was isolated using a Quick RNA isolation kit (Bioteke Corporation, Beijing, China), characterized on a 1% agarose gel, and examined with an RNA Nano 6000 assay kit on a Bioanalyzer 2100 system (Agilent Technologies, Santa Clara, CA, USA). Construction of the sequencing libraries and RNA-seq were performed by the Biomarker Biotechnology Corporation (Beijing, China). mRNA was purified from total RNA using poly-T oligo-attached magnetic beads. Fragmentation was conducted using divalent cations under elevated temperatures in NEB next first-strand synthesis reaction buffer (5×). The first-strand cDNA was synthesized using the random hexamer primer and M-MuLV Reverse Transcriptase (RNase H-). The second-strand cDNA synthesis was subsequently performed using DNA Polymerase I and RNase H. The remaining overhangs were converted into blunt ends via exonuclease/polymerase activities. After adenylation of the 3’ ends of the DNA fragments, NEB next adaptors with a hairpin loop structure were ligated to prepare for hybridization. To select cDNA fragments (preferentially 250–300 bp in length), library fragments were purified with an AMPure XP system (Beckman Coulter, Beverly, MA, USA). Then, PCR amplification was performed to enrich the purified cDNA template. Finally, 30 libraries were sequenced using an Illumina NovaSeq platform and 150-bp paired-end reads were generated^56,57^.

### Transcript profiles and annotation

Reference genome and gene model annotation files were downloaded directly from the genome website (https://www.ncbi.nlm.nih.gov/assembly/GCF_000801105.1/#/st). An index of the reference genome was built, and paired-end clean reads were aligned to the reference genome using Hisat2 v2.0.5^58^. The mapped reads of each sample were assembled using StringTie v1.3.3b^59^ in a reference-based approach. FeatureCounts v1.5.0-p3^60^ was used to count the read numbers mapped to each gene. Then, the Fragments Per Kilobase of transcript, per Million mapped reads (FPKM) of each gene was calculated based on the length of the gene, and the read count was mapped to this gene. The differential expression analysis of the two groups was performed using the DESeq2 R package v1.16.1^61^. The resulting *P*-values were adjusted using the Benjamini and Hochberg’s approach for controlling the false discovery rate. Genes with an adjusted *P*-value < 0.05 found by DESeq2 were determined to be differentially expressed. Gene Ontology (GO) and KEGG pathway functional enrichment analyses of the DEGs were implemented based on *Arabidopsis* gene sets using the clusterProfiler R package. PCA using the software R3.0.3 (https://www.r-project.org/) was performed to study accession-specific expression patterns.

### Gene validation and expression analysis

To verify the reliability of the RNA-seq results, eight anthocyanin-related genes were subjected to RT-qPCR with specific primers identified using Primer Premier software (Premier, Canada) (Supplementary Table S1). cDNA synthesis and RT-qPCR were performed as previously described^62^. SYBR Green was used for the detection of PCR products. *Actin* was used as an internal control for the normalization of gene expression. Three independent biological replicates with three technical replicates each were analyzed by RT-qPCR to ensure reproducibility and reliability. Relative expression levels were estimated using the 2^−ΔΔCT^ method^63^.

### Integrative analysis of the metabolome and transcriptome

Pearson’s correlation coefficients were calculated for previous metabolome data^36^ and transcriptome data integration by Metware Biotechnology (Wuhan, China). The mean of all biological replicates of each cultivar in the metabolome data and the mean value of expression of each transcript in the transcriptome data were calculated. The fold-change of each pigmented radish accession (TXH, XLM, and ZJL) was calculated for both the metabolome and transcriptome data, and then compared with the control accession (SZB with white skin and white flesh). Finally, the coefficients were calculated from the log2fold change of each metabolite and transcript using Excel (Microsoft Corporation, Redmond, WA, USA). Correlations corresponding to a coefficient with PCC > 0.95 were selected (Supplementary Table S4). Metabolome and transcriptome relationships were visualized using Cytoscape v2.8.2 (https://cytoscape.org/)^64^.

## Supplementary Information

Supplementary Information 1.

Supplementary Information 2.
