# Supplementary material for: Comparative transcriptome analysis reveals key genes associated with pigmentation in radish (Raphanus sativus L.) skin and flesh
Source: Sci Rep. 2021 Jun 1;11:11434. doi: 10.1038/s41598-021-90633-5 (PMC8169917; doi:10.1038/s41598-021-90633-5)
Supplement: Supplementary file 1 — Supplementary Information 1. [file 41598_2021_90633_MOESM1_ESM.docx]

**Comparative transcriptome revealed key genes involved in radish taproots with different skin and flesh colors (*Raphanus sativus* L.)**

**Jifang Zhang^1, 3^*, Jian Zhao^2, 3^, Qunyun Tan^1^, Xiaojun Qiu^1^ and Shiyong Mei^1^***

^1^ Institute of bast fiber crops, Chinese academy of agricultural science; Center for southern economic crops, Chinese academy of agricultural science, Changsha, China.

^2^ Novogene Bioinformatics Institute, Beijing, China.

^3^ These authors contributed equally to this work.

**^*^** Correspondence author: [hbvegbt@163.com](mailto:hbvegbt@163.com), [smilehome@163.com](mailto:smilehome@163.com)


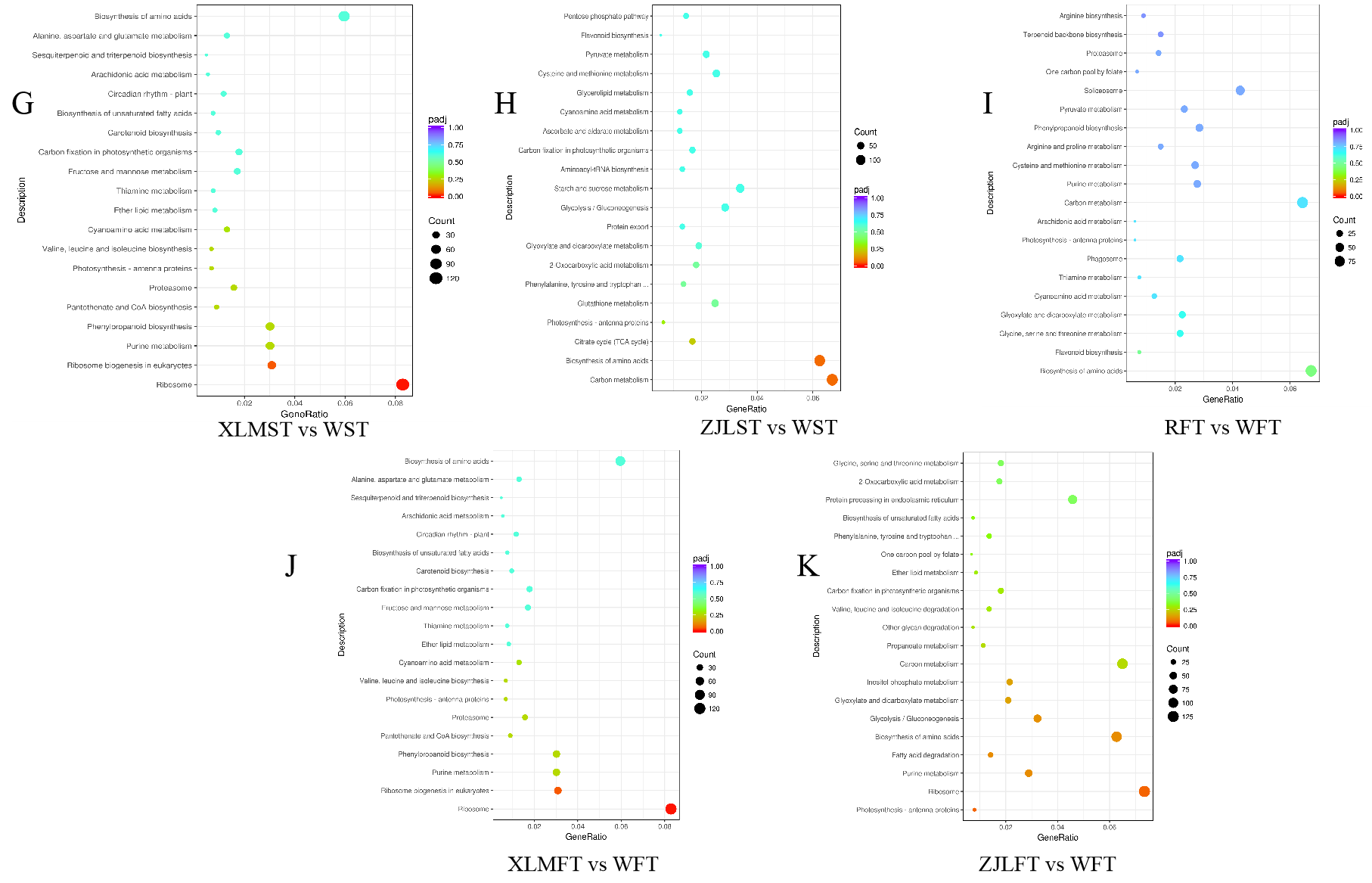

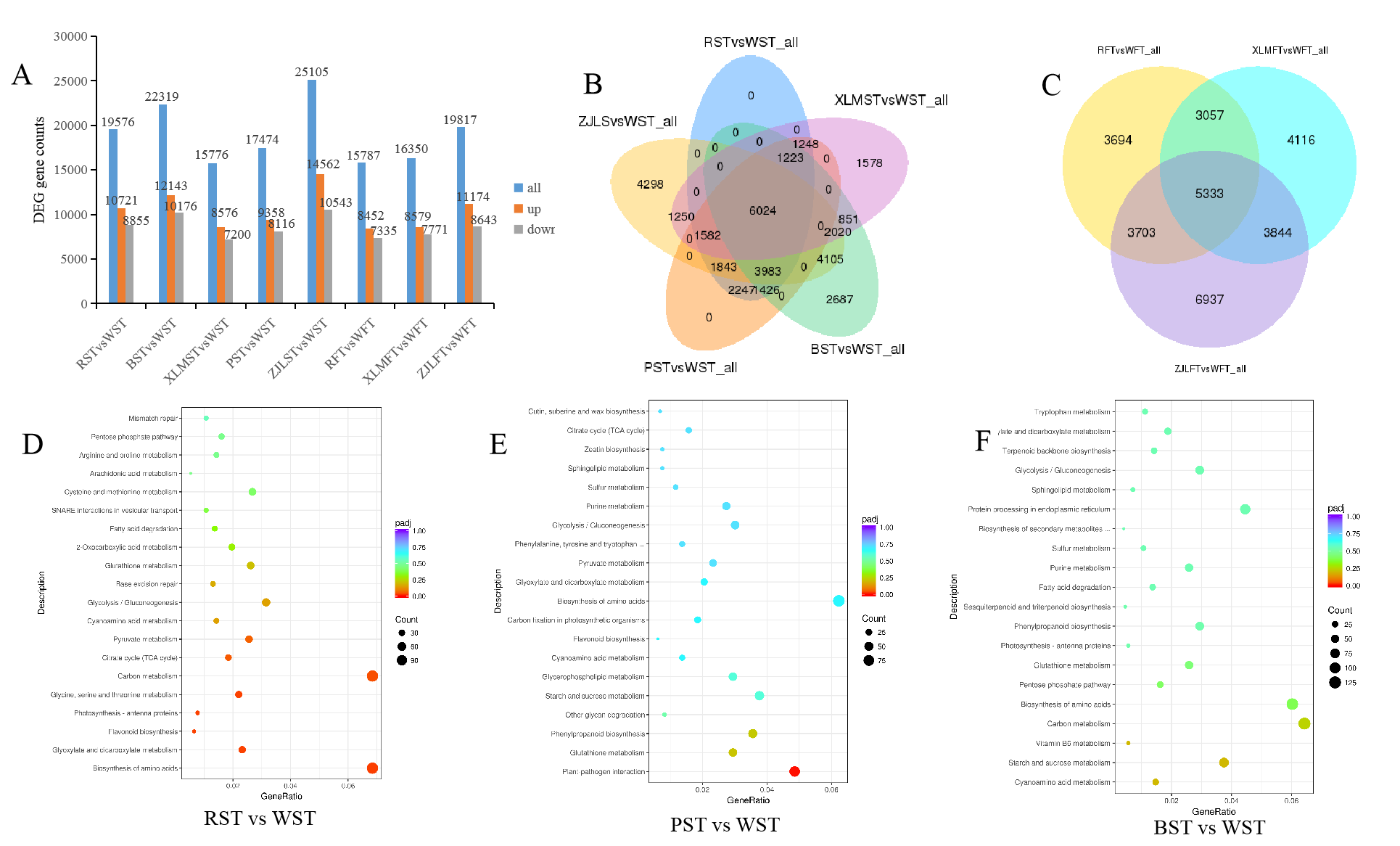


A

B

C

D

E

F

G

H

Figure S1. The top 20 KEGG pathway^(37)^ enriched DEGs in the skin (A–E) and flesh (F–H) of radish taproots.
